# Development and pilot testing of HEXORR: Hand EXOskeleton Rehabilitation Robot

**DOI:** 10.1186/1743-0003-7-36

**Published:** 2010-07-28

**Authors:** Christopher N Schabowsky, Sasha B Godfrey, Rahsaan J Holley, Peter S Lum

**Affiliations:** 1 Center for Applied Biomechanics and Rehabilitation Research (CABRR), National Rehabilitation Hospital, 102 Irving Street, NW Washington, DC 20010, USA; 2 Veterans Affairs Medical Center, 50 Irving Street NW (151), Washington, DC 20422, USA; 3 Department of Biomedical Engineering, Catholic University, 620 Michigan Ave., NE Washington, DC 20064, USA; 4 Neuroscience Research Center, National Rehabilitation Hospital, 102 Irving Street, NW, Washington, DC 20010, USA

## Abstract

**Background:**

Following acute therapeutic interventions, the majority of stroke survivors are left with a poorly functioning hemiparetic hand. Rehabilitation robotics has shown promise in providing patients with intensive therapy leading to functional gains. Because of the hand's crucial role in performing activities of daily living, attention to hand therapy has recently increased.

**Methods:**

This paper introduces a newly developed Hand Exoskeleton Rehabilitation Robot (HEXORR). This device has been designed to provide full range of motion (ROM) for all of the hand's digits. The thumb actuator allows for variable thumb plane of motion to incorporate different degrees of extension/flexion and abduction/adduction. Compensation algorithms have been developed to improve the exoskeleton's backdrivability by counteracting gravity, stiction and kinetic friction. We have also designed a force assistance mode that provides extension assistance based on each individual's needs. A pilot study was conducted on 9 unimpaired and 5 chronic stroke subjects to investigate the device's ability to allow physiologically accurate hand movements throughout the full ROM. The study also tested the efficacy of the force assistance mode with the goal of increasing stroke subjects' active ROM while still requiring active extension torque on the part of the subject.

**Results:**

For 12 of the hand digits'15 joints in neurologically normal subjects, there were no significant ROM differences (P > 0.05) between active movements performed inside and outside of HEXORR. Interjoint coordination was examined in the 1^st ^and 3^rd ^digits, and no differences were found between inside and outside of the device (P > 0.05). Stroke subjects were capable of performing free hand movements inside of the exoskeleton and the force assistance mode was successful in increasing active ROM by 43 ± 5% (P < 0.001) and 24 ± 6% (P = 0.041) for the fingers and thumb, respectively.

**Conclusions:**

Our pilot study shows that this device is capable of moving the hand's digits through nearly the entire ROM with physiologically accurate trajectories. Stroke subjects received the device intervention well and device impedance was minimized so that subjects could freely extend and flex their digits inside of HEXORR. Our active force-assisted condition was successful in increasing the subjects' ROM while promoting active participation.

## Background

Cerebral vascular accident, or stroke, remains the leading cause of adult disability and it is estimated that there are nearly 800,000 stroke incidents in the United States annually [1]. Though stroke causes deficits in many of the neurological domains, the most commonly affected is the motor system [2]. Nearly 80% of stroke survivors suffer hemiparesis of the upper arm [3] and impaired hand function is reported as the most disabling motor deficit [4]. Currently, even following extensive therapeutic interventions in acute rehabilitation, the probability of regaining functional use of the impaired hand is low [5]. Adequate hand function, particularly prehension, is vital for many activities of daily living including feeding, bathing and dressing. Accordingly, there has been much focus on both understanding the mechanisms underlying hand motor function impairment and optimizing hand therapy techniques that elicit greater gains in motor function.

A number of factors that contribute to hand impairment have been investigated. Evidence indicates that hypertonia in finger flexor muscles [6] and weakness in both finger extensor and flexor muscles [7] impair voluntary hand function. The inability of the CNS to activate agonist muscles also plays a large role in hand impairment [8,9]. However, muscle weakness is not uniform between the extensor and flexor muscles [10], and stroke survivors generally tend to regain functional flexion with minimal recovery of extension. These imbalances are related to altered muscle activation patterns where elevated levels of flexor activity occur during intended extension movements [11]. The inability to independently activate muscle groups during extension movements results in co-contraction of antagonistic pairs causing reduced active ROM [12]. However, studies have shown that activity-based repetitive training paradigms that focus on simple flexion and extension finger movements can result in improved grasp and release function [13,14].

The use of rehabilitation robotics to provide motor therapy has shown great potential. Some of the benefits of rehabilitation robotics include introducing the ability to perform precise and repeatable therapeutic exercises, reduction of the physical burden of participating therapists, incorporation of interactive virtual reality systems, and collection of quantitative data that can be used to optimize therapy sessions and assess patient outcomes. Many investigators have focused on developing devices designed to retrain an impaired upper limb [15-19], and robot-assisted therapy is proven to significantly improve proximal arm function [20-25]. However, regaining the ability to 'reach and grasp' allows patients to perform many ADL, providing both functional gains and increased independence. Therefore successful upper arm therapy requires focus on not only the proximal joints of the arm, but also the distal joints found in the hand.

Hand therapy via rehabilitation robotics has received less, but growing, attention. Lately, a number of robots have been developed to provide hand motor therapy. These devices all have similar goals: to develop a training platform that helps patients regain hand range of motion and the ability to grasp objects, ultimately allowing the impaired hand to partake in activities of daily living. However, these devices vary widely in terms of actuated degrees-of-freedom (DOFs), range of motion and design philosophy.

One class of devices uses an "endpoint control" strategy, whereby forces are applied to the distal segments of the digits. HandCARE uses cable loops attached to the ends of each digit. A motor and pulley system apply forces to the digits, and a clutch design allows individual actuation of the fingers and thumb with a single motor [26,27]. The Rutgers Hand Master II is a force-feedback glove powered by pneumatic pistons positioned in the palm of the hand [28] and post-training results reported that chronic stroke patients had clinical and functional gains [29,30]. Amadeo is a commercially available device that provides endpoint control of each of the hand digits along fixed trajectories http://www.tyromotion.com/en/products/amadeo.

Another class of devices is "actuated objects" that can expand or contract. The "haptic knob" uses an actuated parallelogram structure that presents two movable surfaces that are squeezed by the subject [31]. The InMotion Hand Robot uses a double crank and slider mechanism driven by an electric motor, all encased in a cylindrical object [32]. The operation of the motor controls the radius of the cylinder and guides grasping motions.

One disadvantage of endpoint control and actuated objects is limited control of the proximal joints of the fingers, which may lead to physiologically inaccurate joint kinematics, especially in subjects with abnormally increased flexor tone. An alternate approach applies torques to each joint of the finger in a fixed ratio. Two cable-driven devices have been developed that allow for individual control of the fingers and thumb with pulley systems that rest on the dorsal surface of the hand [33,34]. Bowden cables allow the motors to be remotely located reducing the overall weight so these devices can be used in conjunction with arm movements. In a related approach, users don a glove with an air bladder and channels that run along the palmar side of the hand's digits. An electro-pneumatic servovalve is used to regulate air pressure to provide assistance in digit extension. A pilot study of this device resulted in modest functional gains [35]. However the disadvantage of these approaches is that the ratio of torques applied to the joints in a digit is not adjustable. Therefore, abnormal joint kinematics is possible.

A final class of devices is robotic exoskeletons. The joints of the exoskeleton are aligned with the anatomical joints, allowing for proper interjoint coordination between anatomical joints. An example of this approach is the Hand Wrist Assistive Rehabilitation Device (HWARD), a 3 DOF robot that directly controls finger rotation about the metacarpophalangeal joint (MCP), thumb abduction/adduction and wrist extension/flexion [36]. A recent clinical trial reported significant behavioral gains, increases in task-specific cortical activation and a dosage effect where subject gains improved with increased robotic therapy intensity [37]. The Hand Mentor (Kinetic Muscles Inc., Tempe, AZ) is a commercially available exoskeleton device that uses an artificial muscle to simultaneously extend and flex the fingers and wrist [38], but does not actuate the thumb.

Many of the preliminary training studies noted above have resulted in significant clinical and functional gains. These results justify further investigation in the use of rehabilitation robotics for hand motor therapy. In this paper, we introduce a recently developed rehabilitation robot for the hand, the Hand Exoskeleton Rehabilitation Robot (HEXORR). HEXORR is an "exoskeleton" because the robot joints are aligned with anatomical joints in the hand and provides direct control of these hand joints. Unlike other hand exoskeletons which use pneumatic actuators [36,38], HEXORR uses a low-friction geartrains and electric motors. This combination allows for implementation of both position and torque control therapy modes with enough torque capacity to open a hand with high flexor tone. Another advantage is that HEXORR provides physiologically accurate grasping patterns yet is controlled with only two actuators, which contrasts with highly complex designs which incorporate as many as 18 actuators to control the many DOFs of the hand [39]. HEXORR also has been designed to provide nearly full ROM for every digit of the hand. The thumb actuator allows for variable thumb plane of motion to incorporate different degrees of extension/flexion and abduction/adduction. We have also designed a force assistance mode that provides extension assistance based on individual user's needs. This combination of features makes the HEXORR unique compared to other devices under development.

Here, we describe the mechanical design of the exoskeleton as well as the compensation and force assistance algorithms developed to control the device. We also present a pilot study that has served two purposes: to examine HEXORR's ability to allow physiologically accurate extension and flexion movements of the hand's five digits throughout the full ROM and to test a potential hand therapy exercise paradigm designed to promote greater hand extension while maintaining user control of movements in participants that have experienced a stroke.

## Materials and methods

### Mechanical design of the hand exoskeleton

**Figure 1:**
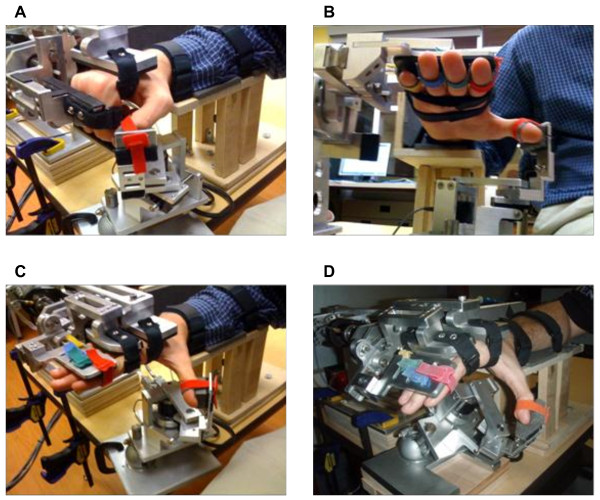
**Pictures of a hand in HEXORR at different postures**. (**A**) The hand flexed. (**B**) Palmar view of the hand in extension, highlighting the Velcro strap arrangement. (**C**) The hand extended, with the thumb in pure extension and (**D**) the hand extended with the thumb in abduction.

HEXORR consists of two modular components that are capable of separately controlling movement of the fingers and thumb (Figure 1). The device acts as an exoskeleton so that the joints of the robot and the user are aligned throughout the allowed ROM. This approach allows for multiple points of contact between the digits and the device, which is critical for properly controlling the kinematic trajectory of the assisted hand movements. General design criteria of this exoskeleton included: 1) allowing the digits full ROM, 2) emulating physiologically accurate kinematic trajectories, 3) providing adjustability to comfortably fit different hand sizes.

The component that actuates the fingers is driven by a four-bar linkage, where the driver link base is aligned with the MCP joints and the driver-coupler joint is aligned with the proximal interphalangeal (PIP) joints. We coupled the rotations of the MCP and PIP joints of all the fingers because it has been shown that joint rotations in one finger closely correlated with adjacent fingers [40]. Although this study showed that the MCP-PIP coordination pattern is slightly less than 1:1, we chose a nearly synchronous rotation of the MCP-PIP joints to maintain the stereotypical spiral finger tip trajectory through 90 degrees of MCP rotation [40]. Three positions of the driver and coupler links were specified in the design: full flexion, full extension, and an intermediate position. An infinite number of 4-bar linkages can be designed that move the driver and coupler through these three positions. The solution space of the four-bar linkage was explored by choosing the coupler-follower joint and graphically determining the ground point of the follower link (Working Model 2D^®^, Design Simulation Technologies, Inc., Canton, MI). This graphical approach led to a general solution capable of generating the desired coupler link path. Using MATLAB^® ^(MathWorks™, Natick, Massachusetts), custom software programs were developed to further analyze and improve the linkage design.

**Figure 2:**
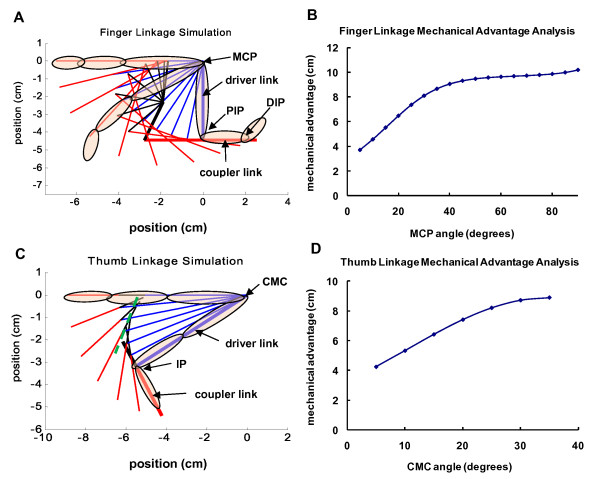
**Linkage motion simulation and force analysis**. (**A**) Finger and (**C**) thumb motion simulation with the initial flexion position linkage configurations bolded and the thumb linkage's slider shaft is shown as a dotted line (green). Finger and thumb images are superimposed at the flexed and extended positions. (**B**) For the fingers, mechanical advantage is output torque at the drive shaft joint that is aligned with the MCP divided by the input force located at the contact point between the linkage and the DIP joints. (**D**) For the thumb, mechanical advantage is the torque at the CMC joint divided by the force at the thumbtip. The x-axis of these plots is the angle of the driver link relative to the fully flexed initial position.

The goal of this analysis was to choose a four-bar linkage design that minimizes the force required by the fingertips to move the linkage through its ROM. We chose this cost function to maximize the backdrivability of the linkage. The lengths of the driver link (length of 3^rd ^digit's proximal phalanx) and the coupler link (length of 3^rd ^digit's intermediate phalanx) are known, and their initial positions are set so that the hand is fully flexed. One hundred possible linkage designs were tested by generating a 2.5 × 2.5 cm grid with a resolution of 0.25 cm centered about the coupler-follower joint position given by the graphical solution. For each candidate coupler-follower joint location, the ground point for the follower was analytically determined that satisfied the three design positions of the driver-coupler links: full flexion, full extension, and an intermediate position. This algorithm also simulated the linkage trajectories by rotating the driver link from full flexion to full extension (90°, 5° per iteration) and solved for the corresponding positions of the other dependent links. Finally, to assess backdrivability, two-dimensional static force analysis was performed per iteration on each of the generated linkage solutions. This analysis simulated the situation when the user is attempting to rotate the static linkage by applying a force at a certain contact point. We focused on the contact point between the dorsal surfaces of the DIPS and the coupler link because it was clear from early prototypes that when the linkage was in certain orientations, large forces were needed at this contact point to rotate the linkage. We assumed that resistance to rotation was due to torque at the drive shaft caused by friction in the geartrain, and all of the other joints in the linkage were frictionless. If the torque at the drive shaft due to the applied force is larger than frictional torque, movement will occur. Thus larger values of shaft torque from external forces would result in higher backdrivability. This analysis assumed that the user's applied force magnitude was constant (1 N) and the direction was normal to the coupler link throughout the ROM. Free-body diagram analysis calculated the torque at the drive shaft needed to statically balance this force in each linkage position. Mechanical advantage was defined as the output torque at the shaft divided by the input force magnitude. The result has units of length and can be interpreted as an effective moment arm between applied force and shaft torque. The final linkage design was chosen by considering linkage kinematic performance (e.g. no singularities, linear coordination between driver link rotation and coupler link rotation), maximizing mechanical advantage and minimizing the range of the mechanical advantage profile over the range of motion. In addition, solutions were not considered if linkage solutions that were nearby spatially had drastically different mechanical advantage profiles. The resulting four-bar linkage design is shown in Figure 2A and the final design performance can be seen in Figure 2B.

The finger component contacts the hand at three locations. To help stabilize the hand inside the device, a hook and loop strap around the palm holds the hand stationary. Also, hook and loop straps are used to attach the proximal and intermediate phalanges to the respective robotic links. To compensate for different hand sizes, the driver and coupler links are adjustable in length. Once the fingers are comfortably strapped to the proper robotic links, the fingers are free to perform extension and flexion movements (Figure 1). Mechanical stops were implemented to ensure patients are never hyper-flexed or hyper-extended during testing sessions. Also, to enhance comfort and reduce fatigue, a custom arm rest with an elbow support was manufactured.

The thumb linkage design was synthesized using similar methods as those used for the finger linkage (Figure 2C). The model simplifies the motion of the thumb's metacarpal and proximal phalanges as a single driver link that rotates about the carpometacarpal joint (CMC). The driver-coupler joint is centered at the thumb IP. The driver-coupler coordination pattern is synchronous, resulting in approximately 20 and 90 degrees of rotation in the CMC and IP joints, respectively. Additional analysis determined that this movement pattern required the coupler-follower joint to move in a nearly straight line. Therefore, the coupler-follower point was placed on a linear bearing, resulting in a crank and slider mechanism. The thumb's distal phalanx is attached to the mechanism's coupler link with a hook and loop strap. As the CMC joint rotates about the driver ground joint, the thumb's metacarpal bone and proximal phalanx closely follow the motion of the driver link. Although it was not necessary to implement in this study, it is possible to also strap the proximal phalanx to the driver link (not shown) to better control the IP and CMC joints. The base of the thumb device is highly adjustable. The mechanism can ascend and descend vertically along a slotted shaft to accommodate varied hand sizes. The base can also be adjusted (tilted and rotated) to increase or decrease the amount of thumb abduction/adduction involved in the exercises. Similar to the finger component, the thumb component allows a large ROM. The final design performance can be seen in Figure 2D.

### Control Hardware and Sensors

The finger four-bar linkage is driven by a direct current, brushless motor (Maxon Motors, Fall River MA) in series with a planetary gear head (reduction ratio 74:1, Maxon Motors, Fall River MA) that is capable of outputting a continuous torque of 9.8 Nm. For position sensing, a digital optical encoder (resolution of 0.002 degrees) is attached to the end of the motor. A second encoder is placed inline between the linkage and the gear head (resolution of .04 degrees). A torque sensor (TRT-200, Transducer Techniques, Temecula CA) is positioned between the motor and the linkage; that is capable of measuring up to 33 Nm of finger flexion/extension torque at a resolution of 0.02 Nm and can serve as both a patient assessment tool and as online feedback to be used in novel therapy techniques.

The thumb component's crank is driven by a FHA mini-series alternating current servo actuator (Harmonic Drive LLC, Peabody, MA) with a harmonic drive gear head (reduction ratio of 100:1, max continuous torque of 11 Nm). This actuator was chosen because of its small housing (60 × 59 × 56 mm) that ensures the thumb component easily fits underneath the finger component. A digital encoder measures shaft angle (resolution of .0005 degrees). A torque sensor (Transducer Techniques, TRT-200) is positioned between the AC servo actuator and the crank.

A single electronic box houses the hardware that controls the motors and interfaces with the torque and position sensors. The motors are controlled by servo drivers operated in torque control mode (Maxon Motors, 4-Q-DC; Accelnet, ACP-055-18). A custom kill-switch can be used to shut down power to both motors. Analog signals from the torque sensors are collected by a data acquisition board (Measurement Computing, PCI-DAS1200). Encoder signals were sampled with a PCI-QUAD04 quadrature encoder board (Measurement Computing).

### Software and Compensation Algorithms

The exoskeleton is controlled with custom software programs developed using the xPC Target^® ^and Stateflow^® ^toolboxes in MATLAB^®^. Because stroke survivors have weakness in the impaired hand, considerable effort was placed on decreasing the torque needed to open and close one's hand inside HEXORR. This was accomplished by increasing the backdrivability of the exoskeleton. Similar to the work outlined in a recent technical note [41], we developed algorithms to model and compensate for the weight and friction (both static and kinetic) of the exoskeleton.

Gravity compensation was modeled by identifying the motor output (current) required to move the linkages throughout the entire ROM at a slow, constant velocity (5°/sec) in both the extension and flexion directions. This produced a current vs. angle profile for each direction. At 1° increments, the values from the extension and flexion profiles were averaged to develop a gravity compensation motor output profile. An interpolation/extrapolation table was created using these data to provide accurate gravity compensation throughout the full movement range of the linkage.

Kinetic friction compensation was modeled through viscosity coefficients. These coefficients were calculated by moving the exoskeleton at different, constant velocities and subtracting the motor output required for gravity compensation. The required motor output (current) increases linearly with velocity (R^2 ^> 0.99) and can be accurately modeled with linear regression equations. These linear models were used to predict and counter velocity-dependent friction. Static friction was estimated by the motor output required to initiate movement after compensating for gravity. This motor output was reduced by a factor of 0.85 to ensure that the linkage does not move when no other forces are applied to the exoskeleton. For this system, increasing the gain beyond 0.85 resulted in over-compensation and caused the robot to move.

The backdrivability of HEXORR was tested by a subject moving the exoskeleton at a constant velocity (40°/sec) with and without compensation. Without any compensation, the torque required to extend the linkages ranged from 0.45 Nm to 0.8 Nm. However, with weight and friction compensation, the required torque was reduced to values no greater than 0.2 Nm and remained constant throughout the movement. On average, the weight and friction compensation algorithms increased HEXORR's backdrivability by more than 66%.

### Safety Measures

Because this exoskeleton is a rehabilitation device designed to interact with individuals that have impaired hands, it is imperative to incorporate both hardware and software safety mechanisms. Mechanical safety stops are positioned so that the fingers and the thumb cannot be hyper-extended when users perform hand movements inside of HEXORR. A kill switch is also implemented so that the experimenter can shut down both motors simultaneously at any time. HEXORR also has software ROM stops. Before each training session, the experimenter manually extends the subject's fingers and thumb asking if the subject feels any pain and also carefully watches for any expressions of discomfort. If the subject cannot tolerate full extension, the experimenter can limit the device's ROM via the graphical user interface. The experimenter can also limit the velocity of the linkages through software controls. Finally, saturation levels are used to ensure that the motor command never exceeds a predetermined threshold.

### Experimental Setup

Nine right-handed, neurologically intact subjects, (aged 23-57 years, mean = 32 ± 12), and five stroke subjects (aged 33-61 years, mean = 53 ± 12) participated in this experiment. All stroke subjects had right hand impairments and handedness was assessed with the ten item Edinburgh inventory [42]. Only subjects that received a laterality quotient of 80% or greater were admitted into this study. All subjects signed an informed consent form prior to admission to the study. All protocols were approved by the Internal Review Board of the MedStar Research Institute.

This pilot study focused on stroke subjects with mild to moderate motor function impairment. For stroke subjects, inclusion criteria required a first ischemic or hemorrhagic stroke occurring more than 6 months prior to acceptance into the study, and trace ability to extend the MCP and PIP joints. Exclusion criteria included excessive pain in any joint of the affected extremity that could limit the ability to cooperate with the protocols, uncontrolled medical problems as judged by the project therapist, and a full score on the hand and wrist sections of the Fugl-Meyer motor function test [43].

Before using the robot, stroke subjects were clinically evaluated (Table 1). Upper extremity movement impairments were evaluated with the Action Research Arm Test [44] and the upper extremity Fugl-Meyer Assessment. Muscle tone was measured at the elbow, wrist and fingers with the Modified Ashworth Scale [45].

**Table 1:** Stroke Clinical Assessments

Measure	All subjects	Subject 1	Subject 2	Subject 3	Subject 4	Subject 5
n	5					
Age (year)		59	61	51	62	33
Gender	1F/4M					
Time post-stroke (months)		14	19	12	300	34
Action Research Arm Test (total score = 57)	22.4 ± 3.2	20	21	21	22	28
Grasp (total score = 18)	6.2 ± 1.1	6	5	6	6	8
Grip (total score = 12)	5.2 ± 1.3	4	4	5	6	7
Pinch (total score = 18)	6.2 ± 0.45	6	6	6	6	7
Gross Movement (total score = 9)	4.8 ± 1.1	4	6	4	4	6
Arm Motor Fugl-Meyer score (total score = 66)	34 ± 7	35	34	35	23	43
Proximal arm subportion (total score = 42)		22	19	20	9	25
Hand/wrist subportion (total score = 24)		12	13	14	13	15
Coordination/Speed (total score = 6)		1	2	1	1	3
Modified Ashworth Spasticity Scale (unimpaired = 0)	1.7 ± 0.3	1 +	1 +	2	1 +	2
Elbow		1 +	1 +	2	1 +	2
Wrist		1 +	1 +	2	1 +	1 +
Finger		1 +	1 +	2	1 +	1 +

Results are mean ± standard error. Subjects received clinical assessment prior to using the robotic device. This pilot study was not intended to provide therapy, so no follow-up assessment was conducted.

Subjects were seated in a chair and their right hand was placed inside HEXORR. The forearm was placed on an arm rest in the neutral position and the table was adjusted so that the elbow was flexed at 90° and the shoulder elevated approximately 45°. An elbow support pad was placed on the posterior side of the upper arm to minimize shoulder retraction and extension. For each subject, the linkages of the exoskeleton were adjusted to fit the size of the hand. The hand was strapped to the device and subjects performed hand movements inside HEXORR for about 30 to 60 minutes. A real-time computer display of their hand's position was available, but in most cases the subjects watched their own hands during the movements.

### Experimental Tasks

Unimpaired subjects performed tasks specifically designed to evaluate HEXORR's ability to produce physiologically accurate hand movements throughout the five digits' ROM. For these tasks, the subjects wore the wireless CyberGlove II^® ^(CyberGlove Systems, San Jose, CA) during movements both inside of and outside of the device. This glove features three flexion sensors per finger, four abduction sensors, a palm-arch sensor, and sensors to measure wrist flexion and abduction. Subjects performed five hand extension/flexion movements throughout the full active ROM outside of the device, five continuous passive extension/flexion movements (finger encoder rotation 0° to 80°, thumb encoder rotation 0° to 20°) in HEXORR, and 10 active-unassisted hand movements inside of the device. Because HEXORR's mechanical safety stops did not allow for hyperextension, subjects were asked not to hyperextend their hand's digits while performing extension movements outside of the device. During the unassisted movements in the device, the motors provided previously described gravity and friction compensation.

**Figure 3:**
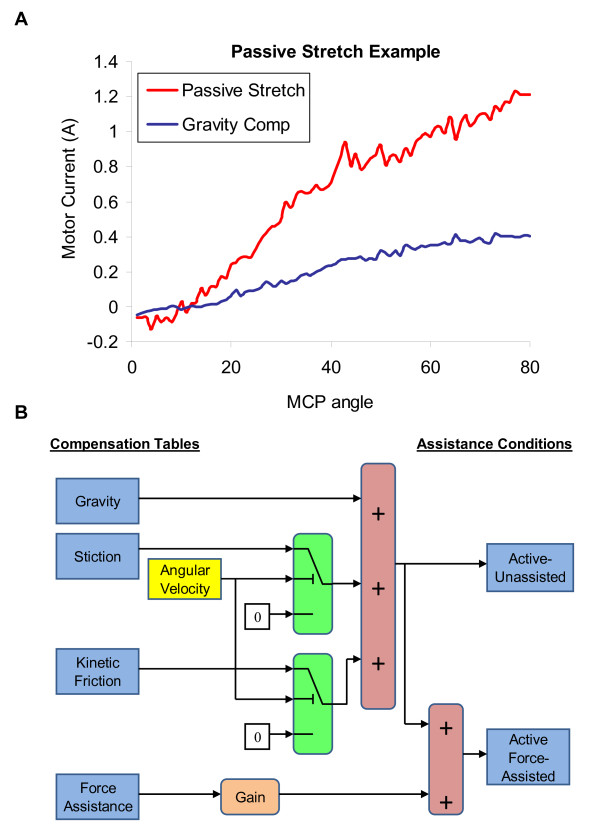
**Assistance condition illustrations**. (**A) **An example of the motor current needed to passively stretch a stroke subject's fingers, compared to gravity compensation. X-axis is the MCP extension angle relative to the fully flexed position. (**B) **Block diagram of the compensation provided for the active-unassisted and active-force assisted conditions. Stiction is provided when -0.1°/sec ≤ angular velocity ≤ +0. 1°/sec. Otherwise, kinetic friction compensation is provided.

Stroke subjects performed hand movements within HEXORR during three different modes: continuous passive movements, active-unassisted extension/flexion and active force-assisted extension/flexion. During the five passive movements, subjects were asked to relax their hand fully as the motors moved their digits throughout a comfortable ROM pre-determined by an occupational therapist (all stroke subjects tolerated full extension of the fingers and thumb). Then, subjects were asked to perform five active-unassisted movements at a self-determined speed. During these movements, motors provided only weight and friction compensation. This mode was also designed to 'catch' any involuntary flexion movements during an intended extension movement. Any unintended flexion movement was halted by the motors, and the exoskeleton was held in place. Subjects were given three attempts to further extend their digits before the experimenter prompted the motors to finish the extension movement. Finally, subjects performed movements during an active force-assisted mode, where subjects received assistance during extension movements. Using data from the previous passive stretching exercises, the mean motor current required to passively extend the subject's digits were tabulated into a position dependent assistance profile. Figure 3A displays an example of the motor current required to passively stretch a stroke subject's hand. This profile was scaled by an adjustable gain and delivered feedforward during the movements. After each extension attempt, the gain was reduced from 1 by increments of 0.2 until the subjects indicated that they were actively opening their hand. Once a proper gain was found, subjects opened and closed their hand five times with this assistance. Figure 3B illustrates a block diagram to further describe the active-unassisted and active force-assisted conditions.

### Data Analysis

Custom software recorded the positions and torques from the robot (*f*_S _= 1 kHz). The encoder signals were digitally differentiated and low pass Butterworth-filtered (*f*_C _= 30 Hz) to yield angular velocity. Torque sensor signals were filtered (*f*_C _= 15 Hz) and biases were removed prior to data analysis. Without a hand in the exoskeleton, the linkages were moved slowly (1°/second) throughout the ROM and the torques were recorded. These torque values were interpolated, averaged and used as position dependent torque sensor bias values. CyberGlove II^® ^data was separately collected using the manufacturer's data acquisition software (*f*_S _= 100 Hz). Calibration of the CyberGlove sensors was performed based on the manufacturer's recommendations. The initiation and cessation of hand movements were defined as 5% of the maximum angular velocity.

For the unimpaired subjects, digit ROM and joint-pair coordination were investigated with the CyberGlove II^® ^data. Active ROM analysis consisted of calculating the difference between the maximum extension and flexion angles in all joints. Joint-pair coordination was assessed for the 1^st ^and 3^rd ^digits under two conditions: outside and inside HEXORR. For the 1^st ^digit, CMC-MCP and MCP-IP joint-pairs were analyzed, and for the 3^rd ^digit, MCP-PIP and PIP-DIP joint-pairs were considered. These pairs were plotted (x axis: proximal joint, y axis: distal joint) and modeled by linear regression. Linearity was measured with the coefficient of determination (R^2^).

For the stroke subjects, the ROM and torque production of the fingers and thumb were compared in the active-unassisted and active force-assisted conditions. The ROM analysis was similar to the unimpaired subject ROM calculation, but by using HEXORR's encoders instead of the CyberGlove II^®^. Average torque values were calculated to investigate the extent of the subjects' voluntary participation during extension movements. Only torque values during exoskeleton movement were considered and torques produced during a pause in motion, caused by hand flexion during a designated extension movement, were removed from the analysis. By convention, positive torque values indicate torque in the extension direction. Therefore, if the average torque during an extension movement was positive, we concluded that the subject performed an active extension movement. Accordingly, if the average torque value was negative, then the provided assistance was too high and the robot pulled the digits open.

Unimpaired subjects' finger active ROM analysis was performed by repeated measures ANOVA with two within subject factors: condition (2: inside and outside of HEXORR) and joint (15 separate joints). All other metrics were statistically evaluated by a paired, two-tailed student t-test.

## Results

**Figure 4:**
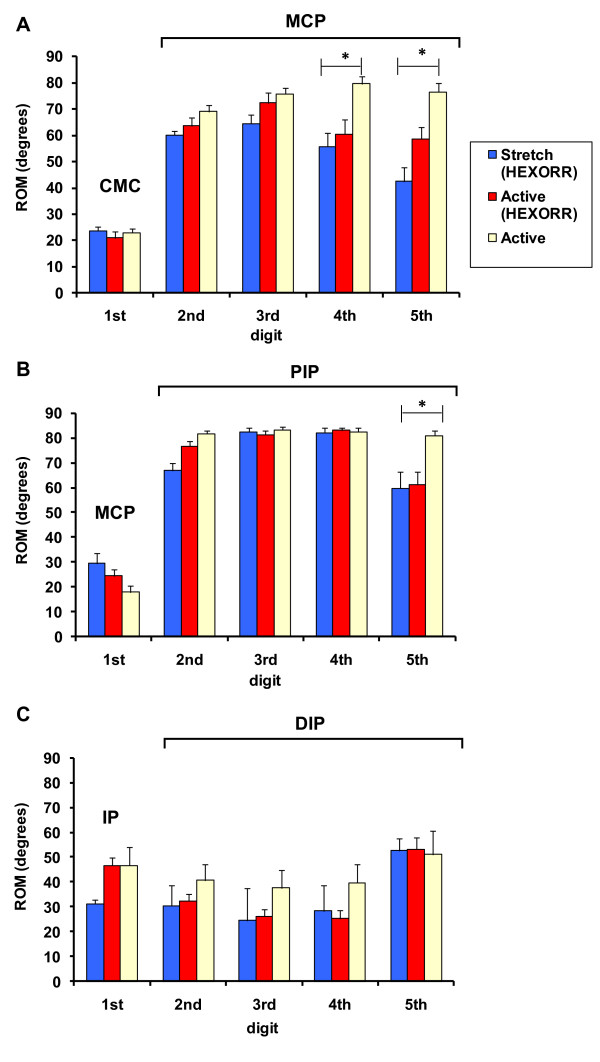
**Unimpaired subjects' ROM**. The mean values of the unimpaired subjects' (**A**) MCP, (**B**) PIP and (**C**) DIP joints for digits 2-5 under 3 conditions: passive stretch, active-unassisted movements inside HEXORR and active movement outside of the exoskeleton. For the first digit, the joints are the (**A**) CMC, (**B**) MCP and (**C**) IP. Twelve of the fifteen tested joints showed no significant ROM differences between active movements outside and inside HEXORR.

Figure 4 illustrates the unimpaired subjects' active ROM (mean ± standard error) under the three conditions: hand movements outside of the exoskeleton, passive stretching and active-unassisted movements inside the exoskeleton. For many of the joints, there were no significant differences between active movements performed inside and outside of the device. Paired t-test analysis showed no significant differences in thumb active ROM. However, the condition factor was significant (F_(1,8) _= 11.6, P = 0.009) for finger active ROM. Post-hoc analysis was performed with Bonferroni corrected paired t-tests. For MCP rotation (Figure 4A), the 4^th ^(difference = 19°, P = 0.017) and 5^th ^(difference = 17°, P = 0.015) digits rotated significantly less inside of HEXORR than outside of the device. The PIP rotation (Figure 4B) of the 5^th ^digit was also significantly less inside of the exoskeleton compared to movements made outside of the device (difference = 23°, P = 0.003). The remaining 12 joints had no significant active ROM differences between movements made inside and outside of HEXORR.

**Figure 5:**
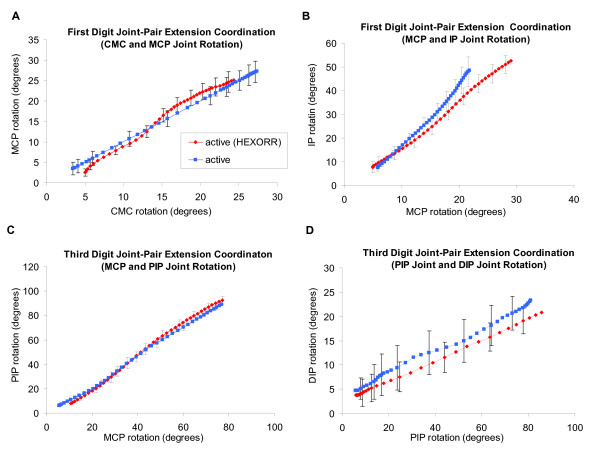
**Joint-pair coordination plots for an unimpaired subject**. Plots display the 1^st ^digit (**A**) CMC-MCP pair (**B**) MCP-IP pair and 3^rd ^digit (**C**) MCP-PIP pair and (**D**) PIP-DIP pair (mean ± standard error). Paired t-tests indicate no significant differences between trajectories performed inside and outside of the exoskeleton. All joint angles are measured relative to the initial fully flexed posture of the hand.

For the 1^st ^and 3^rd ^digits of the hand, mean joint-pair coordination comparisons between active-unassisted extension movements inside HEXORR and those made outside of the device were compared. An example of a subject's joint-pair coordination can be seen in Figure 5. For every subject, the coordination between joint pairs for both the 1^st ^and 3^rd ^digits was highly linear (R^2 ^≥ 0.957) both inside and outside of HEXORR. For the fingers, the average slopes of the MCP-PIP regressions for movements made inside and outside of the device were 1.31 ± 0.24 and 1.17 ± 0.14, respectively and the mean PIP-DIP regression slopes were 0.21 ± 0.1 for movements within HEXORR and 0.15 ± 0.12 for movements outside of the device. For the thumb, the mean slopes of the CMC-MCP regressions for movements made inside and outside of the device were 1.36 ± 0.43 and 1.09 ± 0.38, respectively and the mean MCP-IP regression slopes were 1.99 ± 0.46 for movements within HEXORR and 2.29 ± 0.63 for movements outside of the device. Also, paired t-tests indicated no significant differences between the slopes of the joint-pair coordination plots for the 1^st ^(P > 0.143) and 3^rd ^(P > 0.171) digits. This indicates that performing extension movements with the hand inside HEXORR emulates physiologically accurate extension trajectories.

**Figure 6:**
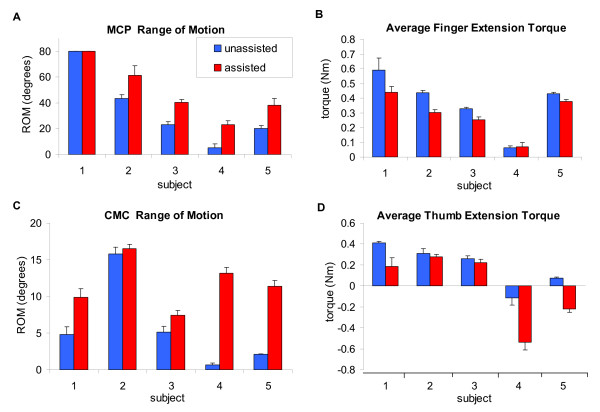
**Stroke subject performance**. (**A**) Finger MCP ROM and (**B**) mean torques and (**C) **thumb CMC ROM and (**D**) mean torques are shown for both the active-unassisted and active force-assisted conditions. The provided assistance increased finger active ROM by 43% and reduced finger extension torque by 22%. For the thumb, active ROM was increased by 24%, reducing thumb extension torque by 30%. For the thumb, the mean torque for Subject 4 and 5 were negative. This indicates that the assistance forces were too high and extended the thumb

Figure 6 summarizes each stroke subject's performance during both the active-unassisted and active force-assisted conditions. Active ROM varied widely on an individual basis (Figures 6A and 6C). The extent of finger extension during the active-unassisted condition ranged from 5° to full extension (80°) at the MCP, and thumb ROM varied between approximately 1° to 16° and 5° to 64° for the CMC and IP, respectively. Average extension torque correlated positively with extension ROM (Figures 6B and 6D). Generally the higher the average torque, the greater the active ROM. The displayed active force-assisted condition values were generated by averaging 5 extension movements while providing assistance with a gain of 0.6. Note that mean thumb extension torques during the active force-assisted condition for Subjects 4 and 5 were negative. This indicates that the provided assistance pulled the thumb open. Accordingly, the thumb data for these two subjects were not considered in the group analysis below. With assistance, the mean active extension ROM increased by 17° ± 4.2° (excluding Subject 1) for the fingers' MCP and PIP; the thumb's CMC and IP increased by 2.6° ± 1.2° and 11.7° ± 3° respectively. The provided assistance increased finger ROM by 43 ± 5%, while reducing the required finger extension torque by 22 ± 4%; thumb ROM was increased by 24 ± 6%, while the required thumb extension torque was reduced by 30 ± 5%.

**Figure 7:**
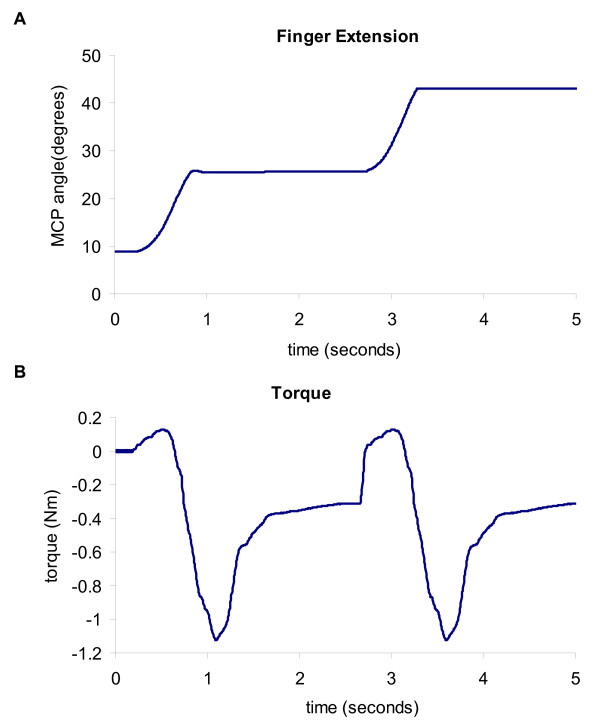
**Extension movement performed by Subject 2**. Flexion motion was halted by the motors and the subject was able to relax the flexors and then further extend the hand's digits.

During both the active-unassisted and active force-assisted conditions, any involuntary flexion movement was halted during a designated extension movement and the stroke subjects were able to try to extend their digits further from this point. Providing this 'flexion catch' greatly increased the active extension ROM for both the fingers and the thumb. On average, the flexion catch feature increased the active ROM by approximately 20° ± 5° for the fingers' MCP and PIP; the thumb's CMC and IP were increased by 5° ± 3° and 22° ± 6° respectively. An example of a stroke subject taking advantage of the 'flexion catch' to increase his fingers' active ROM during the active-unassisted condition can be seen in Figure 7.

## Discussion

We developed a novel exoskeleton to provide hand motor therapy to stroke patients and we conducted a pilot study to test our initial design goals and to evaluate an active force-assistance therapy mode. HEXORR consists of two modular components that are capable of separately controlling the fingers and thumb. This exoskeleton accommodates virtually any hand size and provides extension/flexion assistance for all five digits of the hand through their entire ROM. Our compensation algorithms account for gravity and friction, greatly increasing the device's backdrivability. The main results of our pilot study indicate that, overall, HEXORR was successful in allowing full ROM of the fingers and thumb. Also, the guidance of the linkages maintained physiologically accurate inter-joint coordination throughout the movements. The stroke subjects were capable of active extension during the active-unassisted condition and the active force-assisted condition successfully increased the stroke subject's active ROM while maintaining user control of the movements.

Testing with unimpaired subjects showed that for 12 of the 15 tested hand joints there were no significant ROM differences between hand movements performed inside and outside of HEXORR. Three joints rotated significantly less inside HEXORR, the 4^th ^and 5^th ^digits' MCP and the 5^th ^digit's PIP. We believe that the mechanical stop intended to avoid finger hyper-flexion caused the reduction in the two MCP joints' ROM. This stop was designed to position the 3^rd ^digit's MCP at 90° of flexion (proximal phalanx orthogonal to the palm). Because the machine-hand interface was flat, all of the fingers' proximal phalanges were strapped into this position, resulting in slight misalignment of the MCPs in the shorter digits. Our safety backstop did not allow flexion to 90° in these two MCP joints, thereby reducing their total ROM. It is particularly difficult to strap the intermediate phalanx of the 5^th ^digit to the robot because of differences in digit lengths, and this resulted in a reduced ROM for the 5^th ^digit's PIP. A simple solution calls for a slight redesign so that the 5^th ^digit's phalanges can be individually strapped to the linkage thereby potentially increasing these joints' ROM. The current design of HEXORR is generally successful in producing full ROM of the hand's digits and with a couple of simple design changes this device will allow full ROM for all of the hand's digits.

The stroke subjects were capable of actively extending the hand's digits within HEXORR during the active-unassisted condition. Stroke subjects' ROM varied widely and correlated with their impairment level, as judged by clinical assessment. For instance, Subject 4 performed the worst in the Fugl-Meyer assessment and, accordingly, had the lowest active ROM within HEXORR. All subjects produced torques in the extension direction showing that the active-unassisted condition did not provide overcompensation for gravity and friction. Torque sensor data showed that many subjects unintentionally activated their flexors during extension movements; this typically results in flexing the hand's digits. The 'flexion catch' feature prevented unintended flexion movements during a designated extension movement, and increased the active ROM by approximately 35%. This mechanism is useful because it allows subjects to focus on individually activating their extensor muscles at positions they are normally incapable of reaching. Increasing the digits' active ROM promotes neural activation by creating a larger afferent signal to the brain sensorimotor areas [46].

The assistance provided during the active force-assisted condition further increased the stroke subjects' hand's active ROM. Similar to a previous study [47], we designed this condition so the provided assistance was dependent on the motor current required to passively stretch the subject's digits. This approach directly counters muscle tone, one of the neural mechanisms shown to impede hand extension [6]. Providing assistive forces in the extension direction also inherently helps to counteract the muscle weakness imbalance between the extensor and flexor muscles [9,10]. Generally, torque data showed that, even with assistance, stroke subjects still actively controlled the movements with extension torque. For Subjects 4 and 5, the average thumb torque values were negative, indicating that the assistive forces pulled the thumb open. This is not ideal because it has been shown that providing too much assistance can encourage patients to decrease their own physical effort during therapy [48,49], and impede motor learning [50]. It appears that using the optimal gain in this algorithm will be critical for effective therapy, but the optimal gain will vary across subjects. Therefore, a more sophisticated algorithm is needed to customize the assistance level to the subject. One potential approach is developing an adaptive controller that can adjust the gain of the provided assistance on each trial based on past subject performance [51,52]. This approach has proven successful in prompting both short-term and long-term motor learning while reducing performance error [53,54]. In our application, the adaptive algorithm would use kinematic and torque data from previous trials to adjust the assistance gain to maximize extension ROM while maintaining user control by requiring active extension torque to complete the movements.

Some of the limitations of the HEXORR design can be addressed in future work. Controlling the palmar arch is important in object manipulation and it has been shown that stroke subjects exhibit delayed and incomplete palmar arch modulation during a grasping task [55]. Our device currently has a flat support for attaching to the dorsal surface of the hand and does not assist palmar arch modulation. A potential solution would be selecting a more flexible, pre-shaped (concave) material for the hand support that would allow palmar arch modulation. Similarly, inability to abduct/adduct at the MCP joint can be addressed in future designs by incorporating passive DOFs into the mechanism that allow this motion if the subject is capable. Finally, the current design cannot be used with left hands. We are working on modifications to address this that involve the ability to quickly replace the linkages with mirror-image versions designed for the left hand.

## Conclusions

Our pilot study shows that this device is capable of moving the hand's digits through the entire ROM with physiologically accurate trajectories. We tested stroke patients with mild to moderate motor function impairment who had at least trace ability to extend the fingers. These subjects received the device intervention well and were able to actively extend and flex their digits inside of HEXORR. Our active force-assisted condition was successful in increasing the subjects' ROM while generally promoting active participation. We are currently developing a more sophisticated adaptive active-assistance algorithm to provide the optimal assistance level and profile that promotes motor learning while continuing to challenge the subject's abilities.

## Competing interests

The authors declare that they have no competing interests.

## Authors' contributions

CNS participated in the design of HEXORR, data collection, analysis and interpretation, and manuscript preparation. SBG participated in data acquisition and analysis. RJH participated in subject recruitment and clinical assessment of stroke subjects. PSL participated in the design of HEXORR, data interpretation and manuscript preparation. All authors have read and approved the final manuscript.
